# Effects of an Acute High Dose of Caffeine on Physiological Responses and Performance During a Strength-Focused CrossFit^®^ Workout: A Randomized, Double-Blind, Crossover Study

**DOI:** 10.3390/nu17091419

**Published:** 2025-04-23

**Authors:** Zoe Konidari, Ilias Smilios, Vassilis Mougios, Gregory C. Bogdanis

**Affiliations:** 1 Department of P.E. and Sport Science, Democritus University of Thrace, 69132 Komotini, Greece; zwikonidari@gmail.com (Z.K.); ismilios@phyed.duth.gr (I.S.); 2 Laboratory of Evaluation of Human Biological Performance, School of P.E. and Sport Science at Thessaloniki, Aristotle University of Thessaloniki, 57001 Thessaloniki, Greece; mougios@phed.auth.gr; 3 Department of P.E. and Sport Science, National and Kapodistrian University of Athens, 17237 Athens, Greece

**Keywords:** rating of perceived exertion, lactate, performance, high-intensity functional training

## Abstract

**Background**: Several nutritional strategies have been used to enhance performance in CrossFit^®^ training. This randomized, double-blind, crossover study aimed to investigate the acute effects of caffeine consumption on physiological responses and performance during a strength-focused CrossFit workout. **Methods**: Twelve healthy men, aged 29.2 ± 3.8 years (mean ± SD throughout), with 4.9 ± 1.9 years of CrossFit experience, completed two sessions of a specific CrossFit training program (four rounds of five exercises, 50 s exercise/10 s rest), 60 min after consuming either anhydrous caffeine (7.1 ± 0.7 mg/kg of body mass) or a placebo, aiming to perform as many repetitions as possible. The washout period was at least seven days. At the end of each round, subjective perception of fatigue was recorded using the Borg scale. Blood lactate concentration was measured before and immediately after completing the training session using a portable lactate analyzer. Data were analyzed by factorial ANOVA with repeated measures. **Results**: Caffeine had a negative effect on the number of sit-up repetitions in the fourth round (*p* = 0.012), while it did not affect any other performance parameter, rating of perceived exertion, or lactate concentration compared with the placebo. **Conclusions**: The results of the present study suggest that caffeine consumption does not improve performance in CrossFit training.

## 1. Introduction

CrossFit^®^ is a form of high-intensity training that includes constantly varied functional movements and has gained popularity in recent years, particularly among younger individuals [1]. CrossFit workouts of the day commonly follow two primary formats: “As Many Rounds/Repetitions As Possible” (AMRAP) within a set time frame, and “For Time” workouts aimed at completing a prescribed task as quickly as possible. AMRAP workouts emphasize pacing and endurance, while For Time workouts focus on speed and task completion. Both formats are integral to CrossFit training, each serving distinct fitness goals [1]. Due to its high-intensity nature and the inclusion of loaded exercises, CrossFit presents marked physiological challenges and enhances multiple fitness components, including cardiorespiratory and muscular endurance, strength, power, coordination, balance, and agility [1]. In most CrossFit training programs, the aim is to perform exercises as fast as possible with little or no rest between exercises and sets, which makes it quite challenging by inducing a high cardiorespiratory and metabolic load [2]. CrossFit athletes use several nutritional strategies to improve performance, including caffeine supplementation. However, several of these strategies are used empirically, and their effectiveness in CrossFit performance has not been examined [3].

Caffeine is a substance used widely in sports, as its ergogenic effect has been confirmed for many forms of exercise, such as endurance activities, resistance training, and power-based sports [4,5]. Aerobic capacity benefits most from caffeine intake. Caffeine supplementation exerts an ergogenic effect at doses of 3–9 mg/kg of body mass [6], but guidelines recommend doses of 3–6 mg/kg [5], as higher doses increase the possibility of side effects, such as tachycardia, palpitations, headache, insomnia, anxiety, and gastrointestinal disturbances, and have also been deemed ineffective in inducing further ergogenic effects [7,8]. The timing of consumption depends on the form of the supplement (e.g., capsules or gums) and is usually 10 to 60 min before exercise [4]. It should be noted that, even though the ergogenicity of caffeine is widely accepted, there are many differences between individuals in terms of performance enhancement. This is probably due to different habitual caffeine intake, sex, training level, time of day [4,5], and genetic differences [4,5,9].

The main mechanism of caffeine action is believed to be the stimulation of the central nervous system. Caffeine has a similar molecular structure to adenosine, so it acts as its antagonist, resulting in increased neurotransmitter release, increased recruitment-activation rate of motor units, and pain suppression [4]. Additional putative mechanisms involve its positive effect on muscle contraction due to increased calcium ion release and sodium-potassium pump activity, as well as improved substrate availability [4,10,11].

The ergogenic action of caffeine on various physical abilities, such as aerobic capacity, speed, muscular strength, and endurance, has been confirmed by studies investigating its effect on each one separately [4,5]. However, in exercise formats such as CrossFit, which combine muscle endurance, strength, power, and cardiorespiratory loads, the effects of caffeine have not been investigated sufficiently [1,12], while the limited current evidence shows conflicting results, i.e., either no effect [12,13] or a positive effect on certain aspects of performance or perceived exertion [14,15,16]. Given the positive effects of caffeine on the separate physical abilities required for CrossFit and the limited number of studies that have investigated this topic, the present study aimed to examine the effects of a relatively high dose of caffeine (7 mg/kg body mass) on performance and physiological responses to a demanding CrossFit workout.

## 2. Materials and Methods

### 2.1. Participants

A power analysis (G*Power, version 3.1.9.2; Kiel University, Kiel, Germany) suggested that a minimum sample size of 9 participants was needed to detect a medium effect size (partial eta squared, or η^2^, of 0.06), based on a power of 0.80, alpha of 0.05, and correlation coefficient of 0.5 between repeated measures. Twelve men, aged 22 to 37, who had been training with CrossFit programs for at least 2 years were recruited from CrossFit gyms in the Attica, Greece, region. Individuals with any medical condition that prevented them from performing the exercises of the training program at the maximum of their capacity were excluded. The systematic use of protein, amino acids, and carbohydrate supplements was allowed. All volunteers were coffee/tea drinkers, and their habitual daily caffeine consumption was recorded. Volunteers who consumed caffeine or guarana supplements were excluded unless they agreed to discontinue them for at least two weeks before participating in the study. After receiving a detailed verbal and written explanation of the protocol, the potential risks, and the right to withdraw at any time, the participants provided written informed consent. The study was approved by the Ethics and Research Ethics Committee of the Department of Physical Education and Sports Science of the Democritus University of Thrace (protocol number ∆ΠΘ/ΕH∆Ε/18605/118; approve date 1 October 2021).

### 2.2. Study Design

This was a randomized, double-blind, crossover study. The volunteers performed a CrossFit training session under two different conditions, at least seven days apart, in a random and counterbalanced order. In one condition, they received 7.1 ± 0.7 mg/kg of body mass (mean ± SD throughout) of anhydrous caffeine in tablet form (Pure Caffeine 200 mg tablets by Myprotein, THG Nutrition Limited, Shepherdsville, KY, USA) 60 min before the start of the training session. The aim was to provide 7 mg/kg of body mass of caffeine, but the use of 200 mg tablets resulted in slight deviations from the target dose (i.e., 7.1 ± 0.7 mg/kg of body mass). In the other condition, they received an equal amount of biotin tablets (Myprotein) as a placebo. Because the caffeine and placebo tablets differed in size and shape, the volunteers were told that they were receiving different forms of caffeine supplements.

### 2.3. Anthropometric Measurements

Height was measured using a Soehnle 5003 digital stadiometer (Soehnle Professional GmbH & Co. KG, Backnang, Germany), while body mass and body composition were assessed using an Inbody 270 body composition analyzer (Biospace Co., Ltd., Seoul, Republic of Korea). Volunteers were required to follow a specific preparation protocol before the body composition analysis, which involved proper hydration on the previous day, fasting for 3 h before the measurement, avoiding alcohol for 24 h and large amounts of water or electrolyte-affecting fluids for 3 h before the measurement, as well as refraining from exercise on the day of the measurement. During the measurements, the volunteers were instructed to wear the lightest clothing possible, remove any metal objects, and ensure their feet and palms were dry and clean.

### 2.4. Dietary and Habitual Caffeine Consumption Assessment

The volunteers were instructed to keep a detailed food diary for the two days preceding the first experimental condition and to accurately replicate it before the second condition. The volunteers submitted their food diaries after completing participation in the study. Additionally, before participating, the volunteers answered questions regarding the duration and quality of their sleep over the past two days (very poor, poor, fair, good, or very good), as well as regarding their willingness to perform the exercise on a scale from 0 (no willingness) to 10 (very high willingness).

To assess habitual caffeine consumption, the volunteers were asked to answer a questionnaire through an interview, which investigated the typical consumption of specific caffeine-containing foods and beverages over the past month. Specifically, the consumption of coffee [frequency, type, quantity, dose (single or double), regular or decaffeinated], tea (frequency, type, quantity, loose leaves or tea bags), cocoa (frequency, quantity), chocolate milk (frequency, quantity), chocolate drink (frequency, quantity), energy drinks (frequency, quantity, brand), cola-type soft drinks (frequency, quantity, brand, caffeinated or non-caffeinated), chocolate [frequency, type (milk or dark), quantity], and chocolate-containing foods [frequency, type (e.g., chocolate cookies or cake), quantity] was assessed.

### 2.5. Medical History Information

Information regarding the volunteers’ medical history was collected through personal interviews. This included details on the presence of cardiovascular diseases, hypertension, hypotension, type I or type II diabetes, malignant diseases, gastrointestinal problems, autoimmune diseases, musculoskeletal issues, musculoskeletal injuries, any other health concerns, and smoking habits. Additionally, the volunteers were asked whether they were taking any medication or dietary supplements to check whether they were taking any caffeine through them.

### 2.6. Aerobic Fitness Assessment and Maximum Heart Rate Measurement

The participants were familiarized with the aerobic fitness assessment test in a preliminary session, where detailed explanations were given verbally and in writing. Maximum oxygen consumption (VO_2_max) was estimated using a 20 m shuttle run test [17,18]. Heart rate (HR) was continuously recorded using an HR monitor (Polar H10, Polar Electro Oy, Kempele, Finland), and the highest value at the end of the test was recorded.

The volunteers arrived in the morning at an indoor gymnasium. Before starting the shuttle run test, a warm-up was performed, including slow running for 5 min and another 7 min of exercises including knee-high and heels-back running, dynamic stretches, and activation exercises of the leg muscles. Then, the volunteers completed the first level of the test as a specific warm-up, and after 3 min of rest, they performed the main test. The procedure was as follows: The volunteers ran back and forth over 20 m as many times as possible while keeping the required pace. The running speed was initially 8 km/h, increased to 9 km/h after one minute, and subsequently increased by 0.5 km/h every minute. The test was terminated when the participant did not reach the line by the beep for two consecutive times. VO_2_max was estimated using a table that matched each volunteer’s score, based on age, to a specific value, and his cardiorespiratory fitness level was then assessed as very low, low, satisfactory, moderate, good, very good, or excellent [17,19].

### 2.7. Main Sessions

To ensure the correct execution of the main tests, the participants completed the exercise protocol in a separate familiarization session 7–10 days before the first main test. For each main session, the volunteers arrived at the gym having followed specific preparation instructions. Specifically, they were instructed to avoid intense exercise for 24 h before the test, be hydrated, eat a carbohydrate-rich meal 3–4 h earlier, avoid alcohol for 24 h before the test, and refrain from energy drinks, supplements, or caffeine on the test day. The two main tests were conducted at the same time of day and under similar environmental conditions for each volunteer. After being weighed, the volunteers took either caffeine or biotin capsules, and 40 min later, they began warming up so that the training session would start 60 min after taking the supplement. Before warm-up, the blood lactate concentration was measured using a Lactate Scout 4 automatic lactate analyzer (EKF Diagnostics GmbH, Magdeburg, Germany). After warm-up, the volunteers rested for 3 min and then began the training session.

The total duration of the session was 35 min, consisting of 15 min of warm-up and 20 min of CrossFit exercise (4 rounds of 5 exercises). The volunteers executed five exercises (50 s on, 10 s off) for four rounds, aiming to perform as many repetitions as possible with the correct technique. The five exercises were push-ups, power cleans, front squats, sit-ups, and deadlifts. The power clean, front squat, and deadlift were performed with an external load of approximately 40%, 50%, and 60% of body weight, respectively. The participants were instructed to perform the repetitions as fast as possible, while correct execution was encouraged and emphasized.

Performance was defined as the total number of repetitions per round (i.e., the sum of repetitions in all exercises per round), as well as the number of repetitions performed in each exercise per round. At the end of each round, the rating of perceived exertion (RPE) was recorded using the 6-to-20 Borg scale, and one minute after the end of the workout, the blood lactate concentration was measured again. The HR was monitored using an HR monitor (Polar H10) throughout the workout and averaged over each round. Additionally, after completing the training sessions, the volunteers provided information about possible side effects (tachycardia, palpitations, feelings of anxiety/discomfort, headache, dizziness, gastrointestinal disturbances, etc.).

### 2.8. Statistical Analysis

Caffeine intake and round number or (in the case of lactate) time were the independent variables, while performance, blood lactate concentration, HR (as a percentage of maximum), and RPE were the dependent variables. The data were analyzed by a two-way analysis of variance (ANOVA) with repeated measurements of both independent variables. To identify statistically significant pairwise differences where there was a significant interaction or main effect, Student’s *t* test with Bonferroni correction for multiple comparisons was applied. The data are presented as mean ± SD. The significance level was set at *p* < 0.05. The data analysis was performed using SPSS Statistical software v. 23 (IBM Corporation, Armonk, NY, USA).

## 3. Results

### 3.1. Descriptive Characteristics of the Participants

Table 1 presents the descriptive characteristics of the participants. Eight volunteers reported taking some form of dietary supplements (protein, amino acids, omega-3 fatty acids, or glutamine). All were non-smokers.

### 3.2. Overall CrossFit Performance

**Figure 1:**
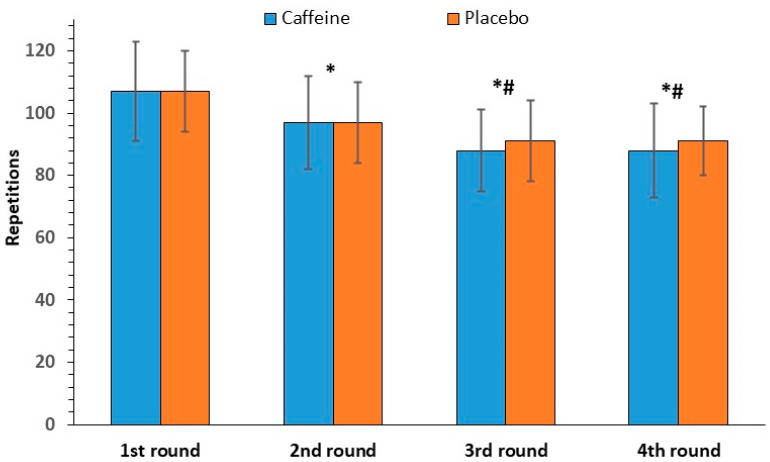
Total repetitions per round (mean and SD) in the caffeine and placebo conditions. * *p* < 0.001 compared to the 1st round. # *p* < 0.001 compared to the 2nd round.

The ANOVA examining the total number of repetitions per round (all exercises summed) revealed no statistically significant interaction between caffeine intake and exercise rounds [F(3, 33) = 2.62, *p* = 0.67]. Regarding the main effects, no significant effect of caffeine intake was found on the total number of repetitions between caffeine and placebo (381 ± 56 and 386 ± 48 repetitions, respectively; F(1, 11) = 0.36, *p* = 0.56], whereas a significant effect of exercise rounds was observed [F(3, 33) = 41.01, *p* < 0.001]. Significant differences were found between all rounds except for the 3rd and 4th. The total number of repetitions per round dropped by 16% from 107 ± 4 in the first round to 90 ± 3 in the last round (Figure 1).

#### 3.2.1. Push-Up Repetitions

**Figure 2:**
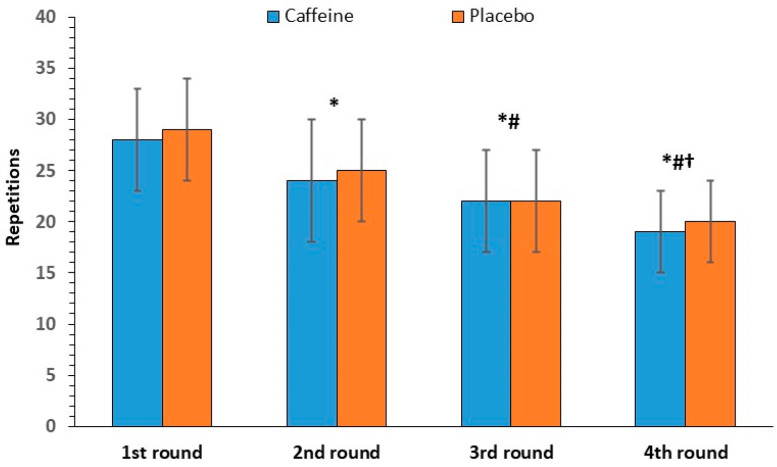
Push-up repetitions per round (mean and SD) in the caffeine and placebo conditions. * *p* < 0.001 compared to the 1st round. # *p* < 0.001 compared to the 2nd round. † *p* < 0.001 compared to the 3rd round.

There was no significant interaction between caffeine intake and round number [F(3, 33) = 0.16, *p* = 0.77] for push-up repetitions. Regarding the main effects, no significant effect of caffeine intake was found [F(1, 11) = 0.31, *p* = 0.59], whereas a significant effect of round number was observed [F(3, 33) = 57.29, *p* < 0.001], which was located between all rounds. Push-up repetitions decreased by 32% from the first to the last round (29 ± 5, to 20 ± 4), the largest drop among the exercises (Figure 2).

#### 3.2.2. Power Clean Repetitions

**Figure 3:**
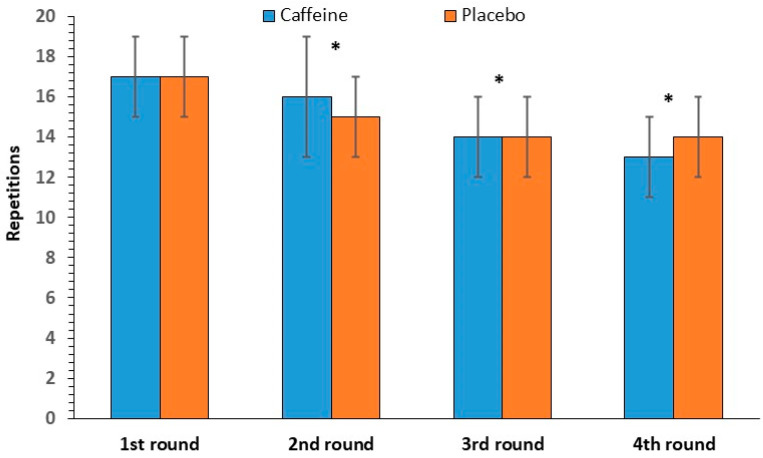
Power clean repetitions per round (mean and SD) in the caffeine and placebo conditions. * *p* < 0.001 compared to the 1st round.

The repeated-measures ANOVA showed no significant interaction between caffeine intake and round number [F(3, 33) = 1.03, *p* = 0.39] in power clean repetitions. There was no significant main effect of caffeine intake [F(1, 11) = 0.06, *p* = 0.81], whereas a significant effect of round number was observed [F(3, 33) = 19.28, *p* < 0.001], which was identified between the first and the subsequent rounds. Power clean performance decreased by 21% from the first round (17 ± 2 repetitions) to the last round (14 ± 2 repetitions, Figure 3).

#### 3.2.3. Front Squat Repetitions

**Figure 4:**
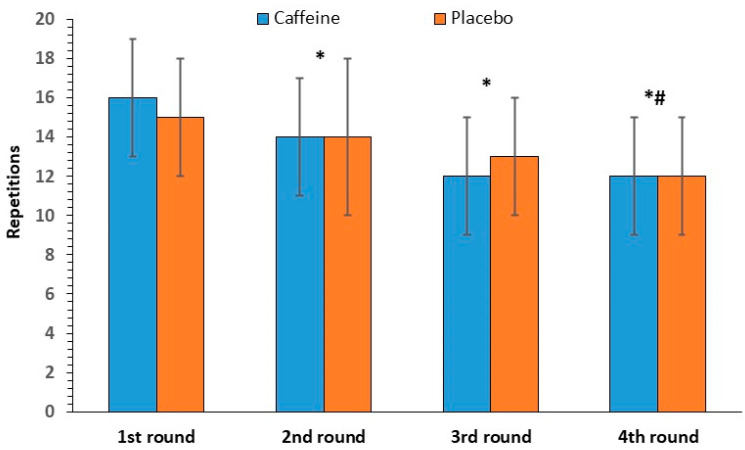
Front squat repetitions per round (mean and SD) in the caffeine and placebo conditions. * *p* < 0.001 compared to the 1st round. # *p* < 0.001 compared to the 2nd round.

The repeated-measures ANOVA revealed no significant interaction between caffeine intake and round number [F(3, 33) = 1.48, *p* = 0.24] for front squat repetitions. Regarding the main effects, no significant effect of caffeine intake was found [F(1, 11) = 0.01, *p* = 0.92], whereas a statistically significant effect of round number was observed [F(3, 33) = 31.58, *p* < 0.001], with decreases from the second round onwards. Front squat performance decreased by 23% from the first to the last round (16 ± 3, to 12 ± 3, repetitions, Figure 4).

#### 3.2.4. Sit-Up Repetitions

**Figure 5:**
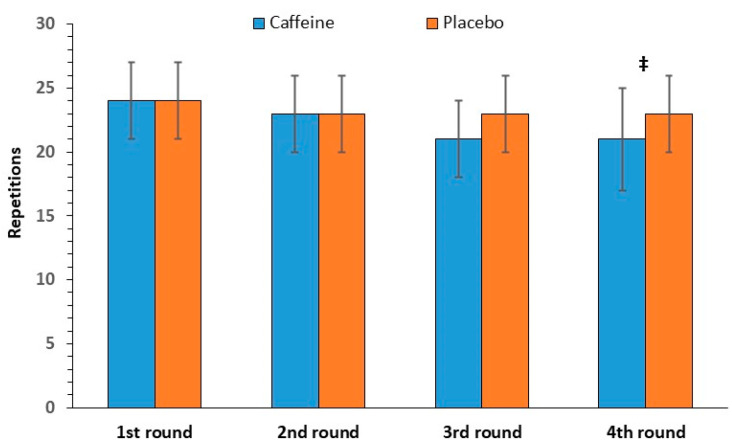
Sit-up repetitions per round (mean and SD) in the caffeine and placebo conditions. ‡ *p* < 0.05 between caffeine and placebo.

The repeated-measures ANOVA showed a significant interaction between caffeine intake and round number [F(3, 33) = 4.24, *p* = 0.01] for sit-up repetitions. Pairwise comparisons revealed a significant difference only in the fourth round (*p* = 0.03, see Figure 5). Specifically, the repetitions were fewer after caffeine intake (21 ± 4) compared with those after the placebo (23 ± 3). It should be noted that sit-up performance was maintained from round 1 to round 4 in the placebo group.

#### 3.2.5. Deadlift Repetitions

**Figure 6:**
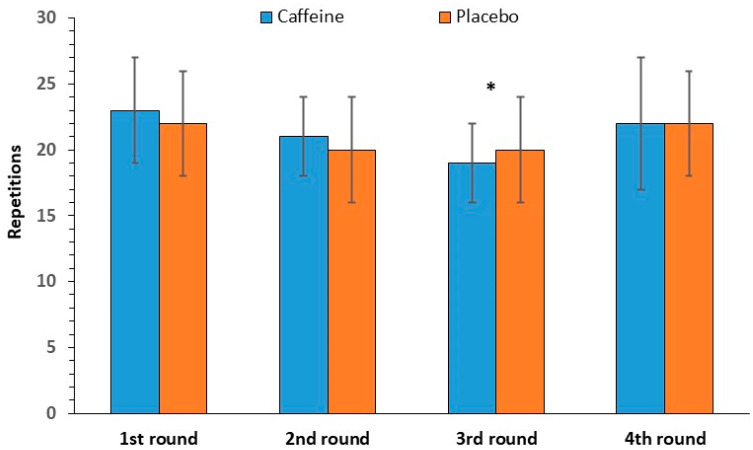
Deadlift repetitions per round (mean and SD) in the caffeine and placebo conditions. * *p* < 0.05 compared to the other rounds.

There was no significant interaction between caffeine intake and round number [F(3, 33) = 0.86, *p* = 0.47] in deadlift repetitions. Regarding the main effects, no significant effect of caffeine intake was found [F(1, 11) = 0.04, *p* = 0.85], whereas a statistically significant effect of round number was observed [F(3, 33) = 8.34, *p* = 0.003], which was located only between the third round and all other rounds (Figure 6).

### 3.3. Blood Lactate Concentration

**Figure 7:**
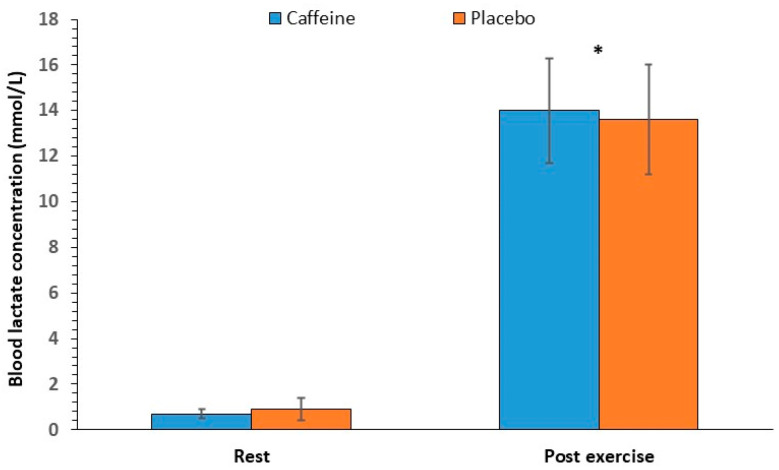
Blood lactate concentration at rest and after exercise (mean and SD) in the caffeine and placebo conditions. * *p* < 0.001 compared to rest.

No significant interaction was found between caffeine intake and time [F(1, 11) = 0.68, *p* = 0.43] in blood lactate concentration, as well as no main effect of caffeine intake [F(1, 11) = 0.17, *p* = 0.69]. However, the main effect of time was observed [F(1, 11) = 519.18, *p* < 0.001], with blood lactate increasing from 0.8 ± 0.1 mmol/L at rest to 13.8 ± 0.6 mmol/L after the completion of the exercise session (Figure 7).

### 3.4. Mean and Peak Heart Rate

**Figure 8:**
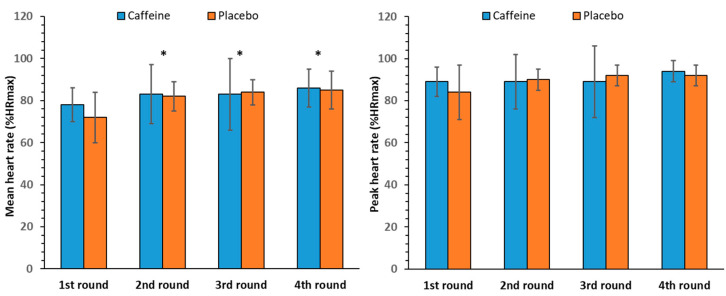
Relative mean (**left panel**) and peak heart rate (HR; **right panel**) per round (mean and SD) in the caffeine and placebo conditions. * *p* < 0.05 compared to the 1st round.

The two-way ANOVA for mean HR showed no significant interaction between caffeine intake and round number [F(3, 33) = 2.16, *p* = 0.16]. Regarding the main effects, no significant effect of caffeine intake was found [F(1, 11) = 0.49, *p* = 0.50], whereas a significant effect of round number was observed [F(3, 33) = 19.24, *p* < 0.001], which was located between the first round and all other rounds. The mean HR increased from 75 ± 3% in the first round to 86 ± 2% in the last round of exercise (Figure 8, left panel).

The ANOVA for peak HR revealed no significant interaction between caffeine intake and round number [F(3, 33) = 1.96, *p* = 0.18]. No significant main effects of caffeine intake [F(1, 11) = 0.07, *p* = 0.80] or round number were noted [F(3, 33) = 3.49, *p* = 0.06. The peak HR reached 93 ± 5% in the last round (Figure 8, right panel)].

### 3.5. Rating of Perceived Exertion

**Figure 9:**
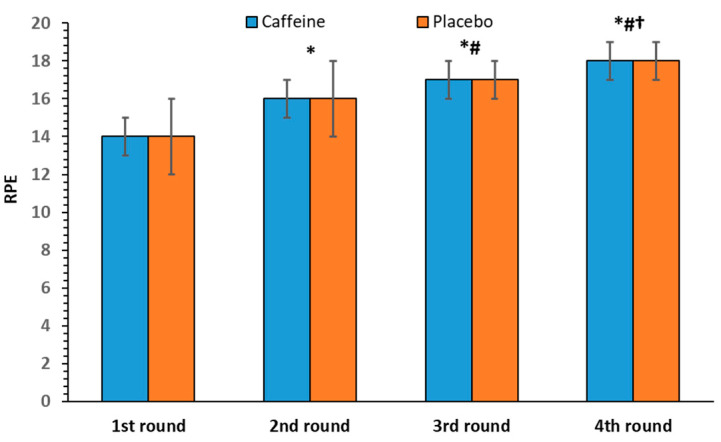
Rating of perceived exertion (RPE) per round (mean and SD) in the caffeine and placebo conditions. * *p* < 0.001 compared to the 1st round. # *p* < 0.001 compared to the 2nd round, † *p* < 0.001 compared to the 3rd round.

The repeated-measures ANOVA revealed no statistically significant interaction between caffeine intake and round number [F(3, 33) = 0.25, *p* = 0.86] in the RPE. No significant effect of caffeine intake was observed [F(1, 11) = 0.31, *p* = 0.59], whereas a significant effect of round number was found [F(3, 33) = 91.24, *p* < 0.001], which was identified across all rounds. Specifically, the RPE scores gradually increased from 14 ± 1 in the first to 18 ± 1 in the last round (Figure 9).

### 3.6. Side Effects of Supplementation

At least one side effect was reported by 67% and 25% of volunteers after caffeine and placebo supplementation, respectively. The main side effect of caffeine was gastrointestinal disturbances (feeling of nausea) in 33.3% of the volunteers (Table 2).

## 4. Discussion

The main finding of the present study was that the intake of 7.1 ± 0.7 mg/kg of body mass of anhydrous caffeine 60 min before performing a CrossFit session had no effect on the total number of repetitions and on the repetitions per round in most of the exercises included in the program. There was only a small (9.5%) decrease in the number of repetitions in the sit-up exercise in the 3rd and 4th rounds. Moreover, neither subjective (RPE) nor measured internal load variables (i.e., HR and blood lactate concentration) were affected by caffeine supplementation.

Similar results were shown in four other randomized, double-blind, crossover studies that investigated the effect of caffeine on CrossFit performance [12,13,14,16]. Specifically, Fogaca et al. [13] reported that the intake of 6 mg/kg 60 min before performing a CrossFit session did not improve performance including as many rounds as possible of double-unders and power snatches in 10 min, with the participants being men with a similar age and CrossFit training experience as those in the present study.

The same conclusion was reached in the study of Stein and colleagues [12], in which twenty men aged 27 ± 6 years, with 4 ± 3 years of experience in CrossFit, performed the “Cindy” program 60 min after consuming 5 mg/kg of caffeine. The “Cindy” program includes five pull-ups, ten push-ups, and fifteen air squats, with the goal of performing as many repetitions as possible in 20 min. Similar results in “Cindy” performance were revealed by the study of Ziyaiyan et al. [16], in which 20 male CrossFit participants, aged 22.3 ± 2.9 years, with at least two years of CrossFit experience, ingested 6 mg/kg of caffeine 50 min before performing the “Cindy” workout.

Glowka and colleagues [14] investigated the effect of three different doses of caffeine (3, 6, and 9 mg/kg) 70 min before a CrossFit session on CrossFit performance, assessed using the Fight Gone Bad test (three rounds × five 1 min exercises with 1 min of rest) and found no effect compared with the placebo. The study involved 26 (10 female and 16 male) moderately trained CrossFit practitioners, who executed three 5 min rounds of five exercises lasting 1 min each: wall ball shots, sumo deadlift high pulls, box humps, push presses, and rowing, aiming to complete as many repetitions (or calories on a rowing ergometer) as possible. The results revealed no significant differences between any caffeine dose and the placebo [14]. On the other hand, Caetano and colleagues [15] found that 6 mg/kg of caffeine improved performance in CrossFit athletes (eight trained men, aged 30.1 ± 6.5 years) when taken 60 min before starting the tests (back squat repetitions to failure with 60% of maximum load) compared with the placebo. We should note that this CrossFit program included only one exercise performed to exhaustion and is thus different from the programs used in the present and other studies [12,13,14,16].

There is limited evidence on the effect of caffeine supplementation on muscular endurance exercises that involve multiple muscle groups with limited rest, such as CrossFit [1,12], with the results being conflicting and inconclusive [20,21,22,23,24,25,26]. According to a meta-analysis [22], caffeine improves muscular endurance by 6–7% when large muscle groups are engaged through repetitions to exhaustion in multiple sets of weightlifting, with fewer than 30 repetitions, a number not comparable to the total repetitions performed in CrossFit programs like the one in the present study. Additionally, even for the number of repetitions of each exercise per round, the results are not comparable, as in our training program, there were insufficient rest intervals, unlike the studies used in the meta-analysis, which also typically involved only isolated exercises and muscle groups.

Several factors could explain the results of the present study regarding performance. Caffeine supplementation may have different effects depending on the individual training level [5]. Specifically, athletes seem to benefit more than physically active individuals, as studies showing positive results in short-duration, high-intensity exercises primarily involve trained athletes. Athletes likely have a stronger motivation to perform strenuous exercises and maintain consistent performance during exercise at regular intervals [5]. The participants in the present study were mainly active individuals, with only two being competitive CrossFit athletes. Indeed, these two athletes performed more repetitions after consuming caffeine than the placebo.

Another factor that could explain the results is habitual caffeine consumption. Regular caffeine consumption seems to reduce the ergogenic effects of acute intake, possibly by altering physiological responses due to increased adenosine receptors [20,21]. However, there is evidence that if the dose administered before exercise is higher than the usual daily intake, caffeine still exerts ergogenic effects [27]. The average daily caffeine consumption of the participants in the present study (136 ± 97 mg) corresponded to 1.7 ± 1.3 mg/kg of body mass, well below the dose administered before exercise. Therefore, it does not seem likely that habitual caffeine consumption explains the results of the present study. In any case, accurately assessing habitual caffeine consumption is challenging due to the varying caffeine content of its sources over time and the fact that our estimation was based on a questionnaire that has not been validated [5,27]. Moreover, there is evidence to suggest that the ergogenic effect of caffeine in doses similar to what was used in the present study (6 mg/kg) in individuals habituated to caffeine may be a combination of biological effects and expectancy as this dose was found to be superior only compared with the control condition and not with the placebo [28].

One noticeable finding of the present study was the slight negative effect of caffeine on the number of sit-ups in the fourth round. Such a negative effect is rare in the literature. Over the past decade, a study by Aedma and colleagues [29] highlighted a detrimental effect on upper body peak power during a repeated-sprint testing protocol. There are also older reports of negative effects on high-intensity interval cycling exercises. The explanation for this finding remains unclear [30,31,32]. Most data on the effect of caffeine on upper-body muscular endurance show either positive [11,21,22,33] or no effects [20,23,24]. In any case, in most studies, upper-body muscular endurance is assessed through bench press, and there are insufficient data on caffeine’s effect on abdominal muscle endurance. From the literature of the past decade, two studies [34,35] evaluated the acute effect of caffeine on abdominal muscular endurance and showed increased sit-up repetitions in adolescent athletes. However, the participants were required to perform as many sit-ups as possible in one minute and only for one set, not within the context of a training program.

A similar approach, i.e., using one to three sets of upper-body exercises, with repetitions to exhaustion and adequate rest intervals, has been used in several studies investigating the effects of caffeine supplementation on upper-body muscular endurance [33]. This approach differs from CrossFit training programs. Additionally, it is possible that the ergogenic effect of caffeine may not be the same across all muscles [36]. It has been suggested that small muscle groups have a limited capacity to increase motor unit recruitment through caffeine intake [20,36]. Since increased motor unit recruitment is one of the mechanisms by which caffeine may enhance performance [37], this could be one reason why a positive effect was not observed. Finally, a factor that might explain this result is that three of the eight individuals who performed fewer abdominal repetitions after caffeine intake experienced gastrointestinal discomfort, which may have affected their performance [8].

The absence of an effect of caffeine intake on the RPE in the present study is consistent with the findings of the other four studies that investigated the potential of caffeine to reduce the RPE in CrossFit [12,13,14,16]. Improving performance by reducing the RPE is a possible mechanism of action of caffeine, as many studies have observed a simultaneous increase in performance and reduction in the RPE following acute caffeine supplementation. In endurance exercises, the reduction in the RPE accounts for 29% of the positive effect of caffeine under submaximal exercise conditions, and studies highlight a positive effect of caffeine on the RPE in resistance exercises too, whether related to muscular strength or muscular endurance [6]. For example, in the study by Grgic and colleagues [20], a 3% increase in the one-repetition maximum in barbell squats was observed along with a 7% reduction in the RPE on the Borg 6–20 scale. Moreover, studies by Duncan and colleagues demonstrated an improvement in muscular endurance of both the upper and lower body with a simultaneous reduction in the RPE following caffeine administration [38,39].

Nevertheless, most studies have not revealed any effect of caffeine on the RPE in resistance exercises, even when performance improvement was observed [6]. The meta-analysis by Doherty and Smith showed that the RPE decreases following caffeine administration during prolonged endurance exercises but remains unchanged when assessed at the end of an exhaustive exercise [40], as was conducted in the present study. Another possible explanation is the short rest period between exercises, suggesting that caffeine may not be able to overcome the feeling of accumulated fatigue [41]. Finally, although the RPE is usually assessed using the Borg scale [6], there are questions regarding its validity when used in sports like CrossFit, which involve high volumes of muscular endurance, multiple muscle groups, and limited rest durations [42].

Another variable investigated in this study was the blood lactate concentration after the CrossFit exercise program. The failure to detect an effect of caffeine supplementation is consistent with several studies [41,43,44,45]. Moreover, since no improvement in performance was observed, the lack of a difference in lactate between caffeine and placebo seems expected, assuming that a further increase in lactate concentration would indicate an enhancement in the contribution of anaerobic metabolism [41]. On the other hand, many studies show higher blood lactate following caffeine than placebo consumption, accompanied (or not) by an improvement in performance [14,46,47]. Although one of the proposed mechanisms for the higher lactate response following caffeine consumption is the increased anaerobic metabolism, studies found a higher blood lactate concentration during exercise at a fixed load (70% VO_2_max) without a difference in muscle concentration, suggesting that caffeine might inhibit lactate clearance by the liver or inactive muscles or that it might increase lactate release from other tissues [41].

Heart rate responses to the CrossFit exercise protocol were also not affected by caffeine supplementation, with both the mean and peak HR remaining high. This finding is in accordance with the results of Glowka et al. [14] and Ziyaiyan et al. [16]. The literature regarding the effect of caffeine on HR during resistance exercise presents conflicting data, which mainly focus on peak HR during or at the end of exercise, with most studies showing no effect [6,48,49]. Similarly, a meta-analysis conducted by Glaister and Gissane, which investigated the effect of caffeine consumption (3–6 mg/kg) on HR during submaximal exercise (60–85% VO_2_max) lasting 5–30 min, showed no effects at any dose [10]. These results agree with the present study, which involved a high-intensity training program (average HR during exercise 81 ± 8% of maximum HR) [50] lasting 20 min. Finally, given the high exercise intensity, the findings are somewhat expected, as it seems that the effect of caffeine on HR decreases as exercise intensity increases [46,51].

A limitation of the present study is the relatively small sample size, which is nevertheless comparable to sample sizes in other CrossFit studies on caffeine supplementation [12,13], as well as many other studies examining caffeine’s effects on general exercise performance [11]. Additionally, the inclusion of only male participants limits the expansion of the findings to female CrossFit athletes, for whom the rate of caffeine metabolism may be affected by menstrual cycle phases (estrogen levels) or the use of contraceptive medications, leaving the possibility for different effects of caffeine supplementation [5]. Furthermore, the plasma caffeine concentration was not assessed, and there was no analysis of the polymorphism of the genes responsible for caffeine metabolism (ADORA2A and CYP1A2), which appear to influence its ergogenic effects [5]. Finally, a general limitation of studies on CrossFit is that, although these programs are characterized by high intensity with limited rest periods and by the activation of multiple muscle groups, they exhibit variability in aspects such as duration and muscle groups targeted. Therefore, the conclusions drawn from this study should be considered in the context of similar training programs [1].

## 5. Conclusions

The results of the study show that the acute consumption of 7.1 ± 0.7 mg/kg of body mass of anhydrous caffeine 60 min before performing a CrossFit training session did not improve performance and might have had a small negative effect on sit-up performance under conditions of accumulated fatigue, possibly related with gastrointestinal disturbances. Furthermore, caffeine did not affect the RPE and HR during the workout or blood lactate concentration after exercise. Further randomized studies with larger sample sizes are needed to minimize limitations and examine the factors affecting the physiological responses and performance changes following caffeine supplementation in different individuals. Male CrossFit athletes do not seem to benefit from caffeine consumption and should consider the possible side effects in cases where training programs require exercise with small muscle groups, such as sit-ups, under conditions of accumulated fatigue.

## Figures and Tables

**Table 1:** Descriptive characteristics of the study participants (*n* =12, mean ± SD).

Age (years)	29.2 ± 3.8
Weight (kg)	80.1 ± 7.6
Height (cm)	175 ± 5
BMI (kg/m^2^)	26.3 ± 2.0
Body fat (%)	14.4 ± 4.9
Lean body mass (kg)	39.3 ± 3.8
Years of CrossFit experience	4.9 ± 1.9
Daily caffeine consumption (mg)	136 ± 97
VO_2_max (mL/kg/min)	43.1 ± 4.9

**Table 2:** Side effects of caffeine and placebo supplementation. Data are presented as numbers of participants and frequency (%).

	Caffeine	Placebo
Tachycardia	2 (17%)	0 (0%)
Palpitations	2 (17%)	1 (8%)
Dizziness	3 (25%)	2 (17%)
Gastrointestinal Disorders	4 (33%)	0 (0%)

## Data Availability

The original data presented in the study are openly available in FigShare at https://doi.org/10.6084/m9.figshare.28426292.
